# Evaluation of maxillary and mandibular growth patterns with cephalometric analysis based on cervical vertebral maturation: A Japanese cross-sectional study

**DOI:** 10.1371/journal.pone.0265272

**Published:** 2022-04-06

**Authors:** Asuka Manabe, Takayoshi Ishida, Eiichiro Kanda, Takashi Ono

**Affiliations:** 1 Department of Orthodontic Sciences, Tokyo Medical and Dental University (TMDU) Graduate School, Tokyo, Japan; 2 Medical Science, Kawasaki Medical School, Okayama, Japan; Thamar University, Faculty of Dentistry, YEMEN

## Abstract

**Background:**

Appropriate maxillofacial growth and development evaluation is important for effective orthodontic treatment. Growth evaluation is based on physiological age determined by individual development, but not chronological age. One strategy for determining physiological age is using the cervical vertebral bone age.

**Objectives:**

This study aimed to clarify the standard size of the upper and lower jawbones in Japanese patients using the cervical vertebral maturation stages (CVMS) as an index and clarify the growth pattern. And to use the cervical spine age as a diagnostic aid in orthodontic treatment.

**Material and methods:**

Random sampling was performed from the outpatients who visited the Orthodontics department, Tokyo Medical and Dental University Dental Hospital, and 400 patients were enrolled before treatment. Lateral cephalometric radiographs were obtained to measure the height and length of the mandible and the maxilla length with cephalometric analysis. Standard values were calculated for each cervical-spine-age group to analyze changes during mandibular and maxillary growth. Furthermore, we compared the differences between males and females. The Kruskal–Wallis test was used to compare cervical-spine-age groups, and the Steel–Dwass test was used for multiple comparisons. The reliability of CVMS was confirmed by calculating the weighted kappa coefficient (κ).

**Results:**

κ for the degree of intra-evaluator agreement and the degree of the inter-evaluator agreement were calculated, and both indicated almost perfect agreement. We found that the distance between the anterior nasal spine (ANS) and posterior nasal spine (PNS) (i.e., ANS–PNS) increased significantly between CVMS II and CVMS III in males. The distance between Articulare (Ar) and Gonion (Go) (i.e., Ar–Go) and the distance between Go and Pogonion (Pog) (i.e., Go–Pog) increased significantly between CVMS III and CVMS IV in males.

**Conclusion:**

The findings suggested that CVMS is a reliable indicator of the growth stage of the maxilla and mandible.

## Introduction

Understanding the growth and development of the maxillofacial skeleton is essential in orthodontics treatment. Specifically, maxillofacial growth evaluation is necessary for establishing a proper diagnosis in orthodontic treatment, making decisions on treatment alternatives, and determining treatment starting time. Orthodontists have adopted the bone age and tooth age to evaluate physiological age growth, which is more accurate than chronological age growth. There have been numerous reports on the use of carpal bones for bone age evaluation because they contain multiple bone nuclei, can be radiographed easily, and require a smaller exposure dose [1–3]. An alternative to this method is the cervical spine maturation method (CVM), which evaluates the shape of the cervical spine obtained from lateral cephalometric radiographs. In addition, there are various methods to evaluate tooth age: Hellman’s tooth age, which is based on the eruption status; the Demirjian method [4], which evaluates the degree of calcification in seven permanent teeth from panoramic radiographs; and the Cameriere method [5], which evaluates the degree of root formation.

The cervical spine consists of seven vertebrae, and it supports the skull. Ossification of the cervical spine continues from the embryonic stage into adulthood [6]. The original CVM was proposed by Lamparski et al [7] in 1972, and in 2002, Baccetti et al. developed an improved version of the CVM to facilitate staging [8].

Previous research has reported that CVM correlates with carpal bone age assessment and is also suitable for mandibular growth assessment. The mandibular bone’s size and timing of growth are important to achieve good intermaxillary relationships in orthodontic treatment [9, 10]. However, to the best of our knowledge, only a few previous reports have investigated the growth of maxilla and mandible [11–14] in the Japanese population with an unclarified growth pattern. Furthermore, no previous research measured the size of both the maxilla and mandible simultaneously.

Therefore, the purpose of this study was to (1) calculate standard values for the mandibular height and length and maxillary length with cephalometric analysis, (2) evaluate the growth pattern of maxillary and mandibular height and length, and (3) assess the usefulness of the CVMS for growth assessment.

## Methods

### Study design

This cross-sectional study was conducted using a random sample from the patients who visited the outpatient department of the Department of Orthodontics, Tokyo Medical and Dental University Dental Hospital.

### Ethics statement

This cross-sectional study was approved by the Ethics Committee of Tokyo Medical and Dental University Dental Hospital (D2021-026). Before participation in the study, patients and their guardians provided written informed consent in accordance with the research protocol approved by the Institutional Review Board.

### Participants

The inclusion criteria were patients of Japanese descent, aged from 6 to 20 years old. Random coefficients were assigned to 4,000 patients, and samples to be measured were randomly selected. Furthermore, random sampling was repeated until the sample size in each CVMS group was achieved [15]. Eighty patients were randomly selected and allocated to each group based on the maturation stage, with 400 patients included in the study. The exclusion criteria were: patients with syndromes that could influence maxillofacial morphology.

### Cervical Vertebral Maturation Stages (CVMS)

**Fig 1:**
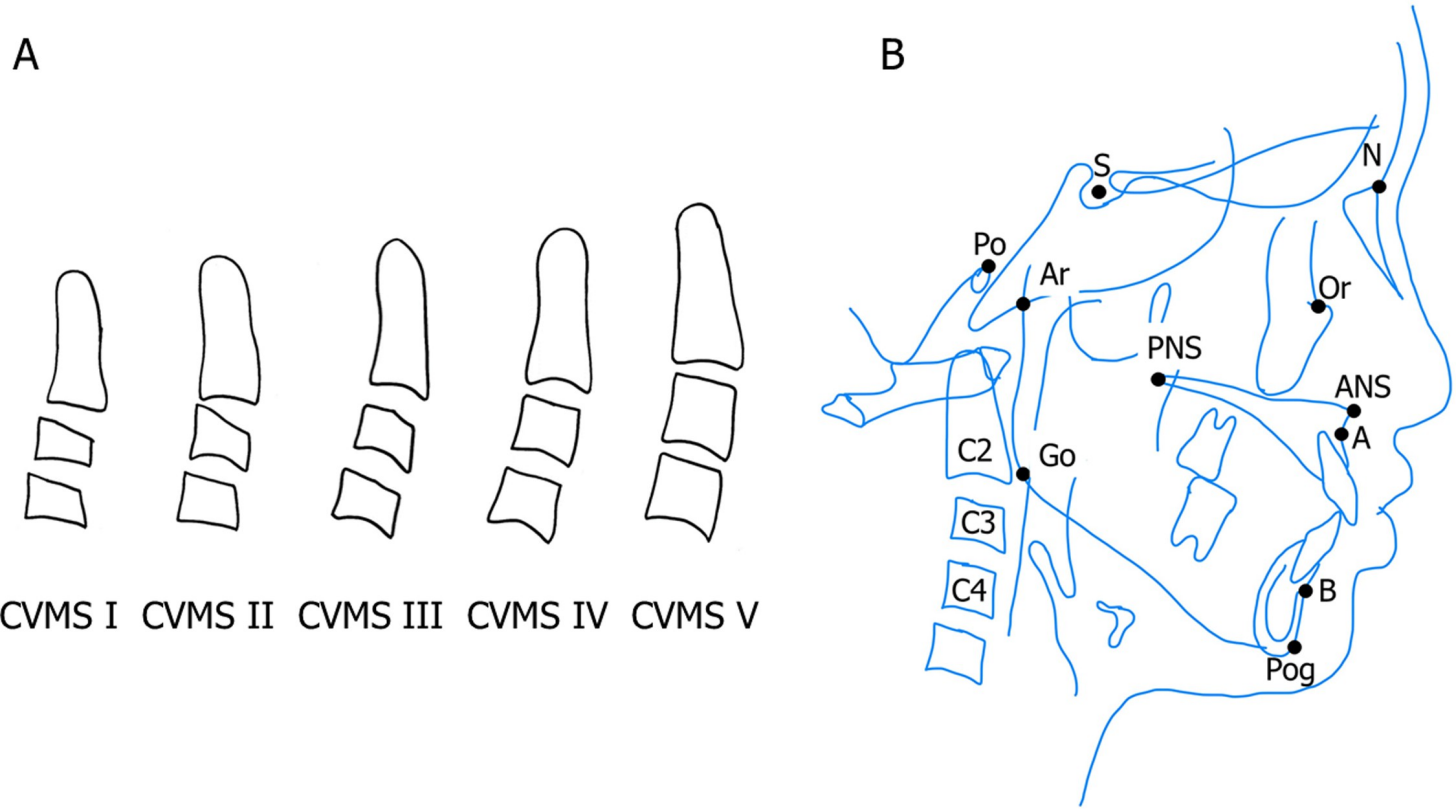
**A.** Definitions of CVMS. CVMS I: The lower borders of three vertebrae are flat. CVMS II: Concavities at the lower borders of both C2 and C3 are present. CVMS III: Concavities at the lower borders of C2, C3, and C4 are present. CVMS IV: At least one of the bodies of C3 and C4 is squared in shape. CVMS V: At least one of the bodies of C3 and C4 is rectangular vertical in shape. **B.** Cephalometric landmarks used to construct the two linear measurements and angular measurements analyzed in this study. Linear parameters: ANS-PNS; Distance between ANS and PNS, Ar-Go; Distance between Articulare and Gonion, Go-Pog; Distance between Gonion and Pogonion. Angular parameters: FMA; Mandibular plane to Frankfort-Horizontal plane, SNA; Sella-Nasion to Point A, SNB; Sella-Nasion to Point B.

The study groups were based on the CVMS I-V. In CVMS I, second (odontoid process, C2), third (C3), and fourth (C4). The lower ends of the cervical vertebrae are all flat. C3 and C4 are trapezoidal in shape. In CVMS II, a dent is present at the bottom of C2 and C3. C3 and C4 are trapezoidal or horizontally long rectangles. In CVMS III, indentations are present on the lower edges of C2, C3, and C4. C3 and C4 are rectangular in shape. In CVMS IV, C3 or C4 is square-shaped. Moreover, the other cervical vertebrae are still long rectangular in the mesiodistal direction. In CVMS V, C3 or C4 has a vertically long rectangle shape. The other cervical vertebrae are also square-shaped [8] (Fig 1A). Descriptive characteristics of the patients are summarized in Table 1.

**Table 1:** Descriptive statistics of patient variables.

	CVMS I				CVMS II				CVMS III				CVMS IV				CVMS V			
	M		F		M		F		M		F		M		F		M		F	
	n = 40		n = 40		n = 40		n = 40		n = 40		n = 40		n = 40		n = 40		n = 40		n = 40	
	mean	S.D.	mean	S.D.	mean	S.D.	mean	S.D.	mean	S.D.	Mean	S.D.	mean	S.D.	mean	S.D.	mean	S.D.	mean	S.D.
	(median)		(median)		(median)		(median)		(median)		(median)		(median)		(median)		(median)		(median)	
age[Y]	10.08	3.14	9.85	3.01	11.06	3.13	10.70	3.04	12.16	1.82	11.72	3.06	16.06	3.10	15.22	2.89	17.29	3.48	16.96	3.37
	9.33		9.25		9.33		9.17		12.13		10.75		16.46		15.71		18.50		18.54	
FMA[°]	29.79	4.84	29.47	5.52	26.45	5.06	29.01	5.54	28.03	5.20	27.00	4.56	26.85	4.94	29.11	4.55	30.22	6.92	31.01	7.09
	30.05		29.30		30.00		28.00		27.70		27.05		27.35		28.80		29.70		32.35	
SNA[°]	79.99	3.22	81.77	3.19	80.61	3.22	81.07	3.39	80.97	3.86	81.64	3.19	81.11	3.39	79.41	3.64	81.48	3.34	80.93	2.97
	80.25		82.25		80.15		82.25		81.66		82.15		81.05		79.35		81.30		81.05	
SNB[°]	76.73	3.44	76.95	3.90	76.78	3.58	77.37	4.16	76.59	4.31	78.59	3.65	79.67	4.60	76.31	4.61	80.19	4.74	78.49	4.87
	76.85		76.65		76.90		77.10		76.10		78.22		79.05		75.30		80.05		78.55	

S.D., Standard deviation; FMA, Mandibular plane to Frankfort-Horizontal plane; SNA, Sella-Nasion to Point A; SNB, Sella-Nasion to Point B; CVMS, cervical vertebral maturation stages.

### Sample size calculations

We calculate sample size using G*Power software (latest ver. 3.1.9.7; Heinrich-Heine-Universität Düsseldorf, Düsseldorf, Germany). The a priori sample size estimation, performed at a 5% level of significance (α = 0.05), with a power of 80%, revealed that a minimum of 40 subjects were necessary per age group.

### Outcome variables

Lateral cephalometric radiographs taken before initiating the orthodontic treatment were traced, and measurement points were plotted (Fig 1B). Variables measured on each lateral cephalometric radiograph are shown in Table 2. All lateral cephalometric radiographs were acquired in accordance with international standards. ANS-PNS was measured as an evaluation of the horizontal diameter of the maxilla, Ar-Go was measured as an evaluation of the vertical diameter of the mandible, and Go-Pog was measured as an evaluation of the horizontal diameter of the mandible [9, 10, 16, 17]. The WinCeph ver.9 (Rise Corp., Tokyo, Japan) software was used to perform the measurements.

**Table 2:** Definitions of measurements variables. Symbol.

Symbol	Description	Definition
S	Sella	The midpoint of the pituitary fossa
N	Nasion	The most anteroinferior point of frontal nasal suture
A	Point A	The deepest point on the curvature of the surface of the maxillary bone between ANS and the alveolar crest of the maxillary central incisor
B	Point B	The deepest point of the curved part of the mandibular alveolar process point
ANS	Anterior Nasal Spine	The cutting edge of anterior nasal spine
PNS	Posterior Nasal Spine	The cutting edge of the posterior nasal spine
Ar	Articulare	Drafting intersection of mandibular process posterior margin and external skull base
Go	Gonion	plotting intersection of the tangents of the mandibular ramus and body
Pog	Pogonion	The most prominent point of the mandibular chin ridge

### Statistical analysis

One investigator performed the measurement three times, and the average value was used as the measured value; however, the evaluation was verified by another investigator. Furthermore, the population was predicted by restoration extraction using bootstrapping with 1000 iterations. The measurement error in each measured value was calculated using Dahlberg’s formula [18] For statistical analysis, the Kruskal-Wallis test was performed for comparison between the groups, and then multiple tests were performed using the Steel-Dwass test. Additionally, to verify the reliability of the CVMS determination, the weighted Kappa coefficient was calculated based on the degree of intra-evaluator and inter-evaluator agreement. The degree of intra-evaluator agreement was based on two CVM staging decisions made by one evaluator in 3 weeks interval. The degree of inter-evaluator agreement was based on the CVMS determined by each of the two evaluators. SPSS version 25 (Statistical Package of Social Sciences, Chicago, IL, USA) was used for statistical analyzes.

## Results

### Linear comparisons

**Fig 2:**
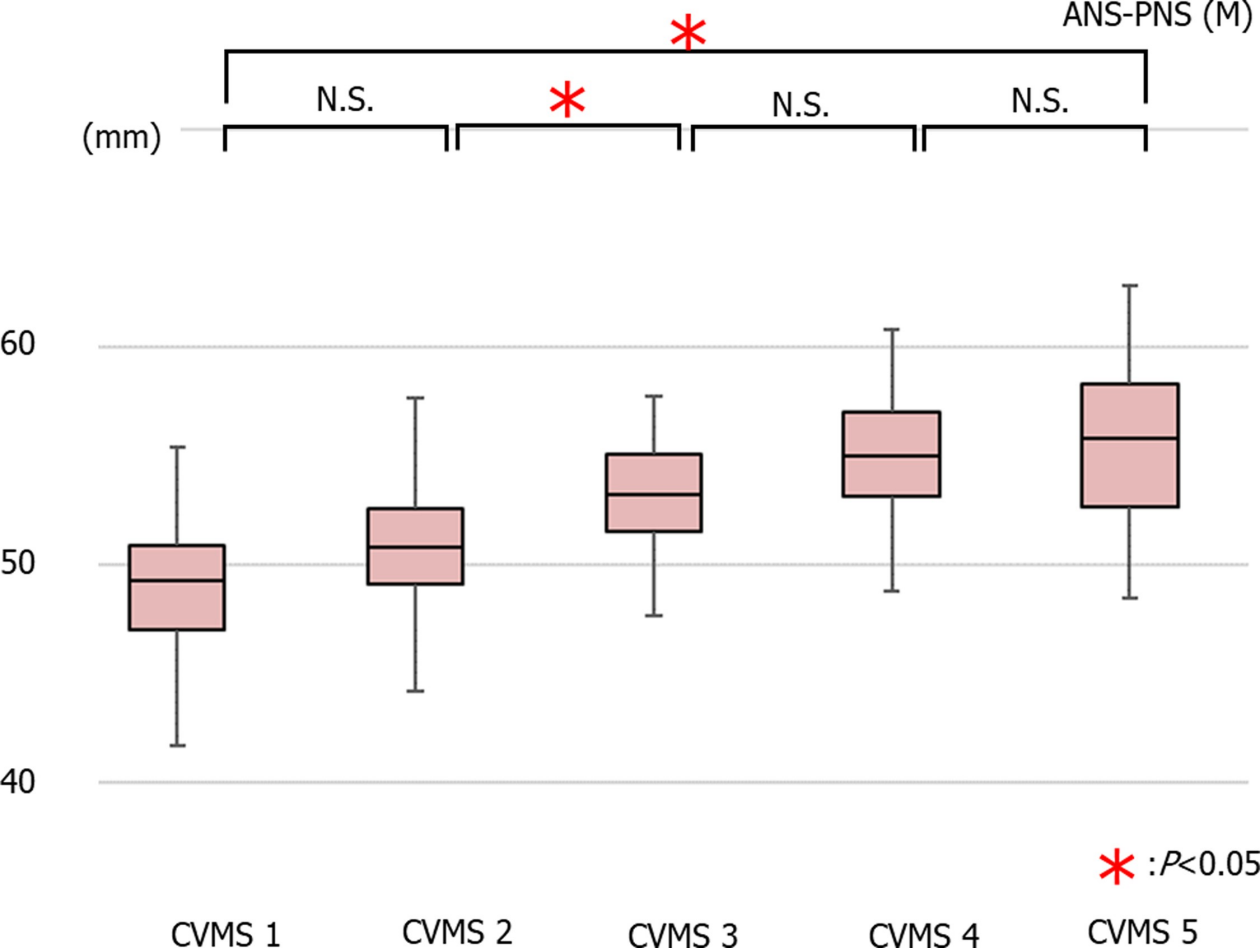
The average values of ANS-PNS in males (*p<0.05).

The average values of the distance between ANS and PNS (ANS-PNS) in males were 49.31 ± 2.74 mm in (cervical vertebral maturation stages) CVMS I Group, 50.76 ± 2.99 mm in CVMS II, 53.11 ± 2.56 mm in CVMS III, 54.95 ± 3.19 mm in CVMS IV, and 55.88 ± 3.42 mm in CVMS V. There was a significant decrease in the ANS-PNS value in CVMS I compared to CVMS III, IV, and V, in CVMS II compared to those in CVMS III, IV, and V, and in CVMS III compared to that in CVMS V (Fig 2).

**Fig 3:**
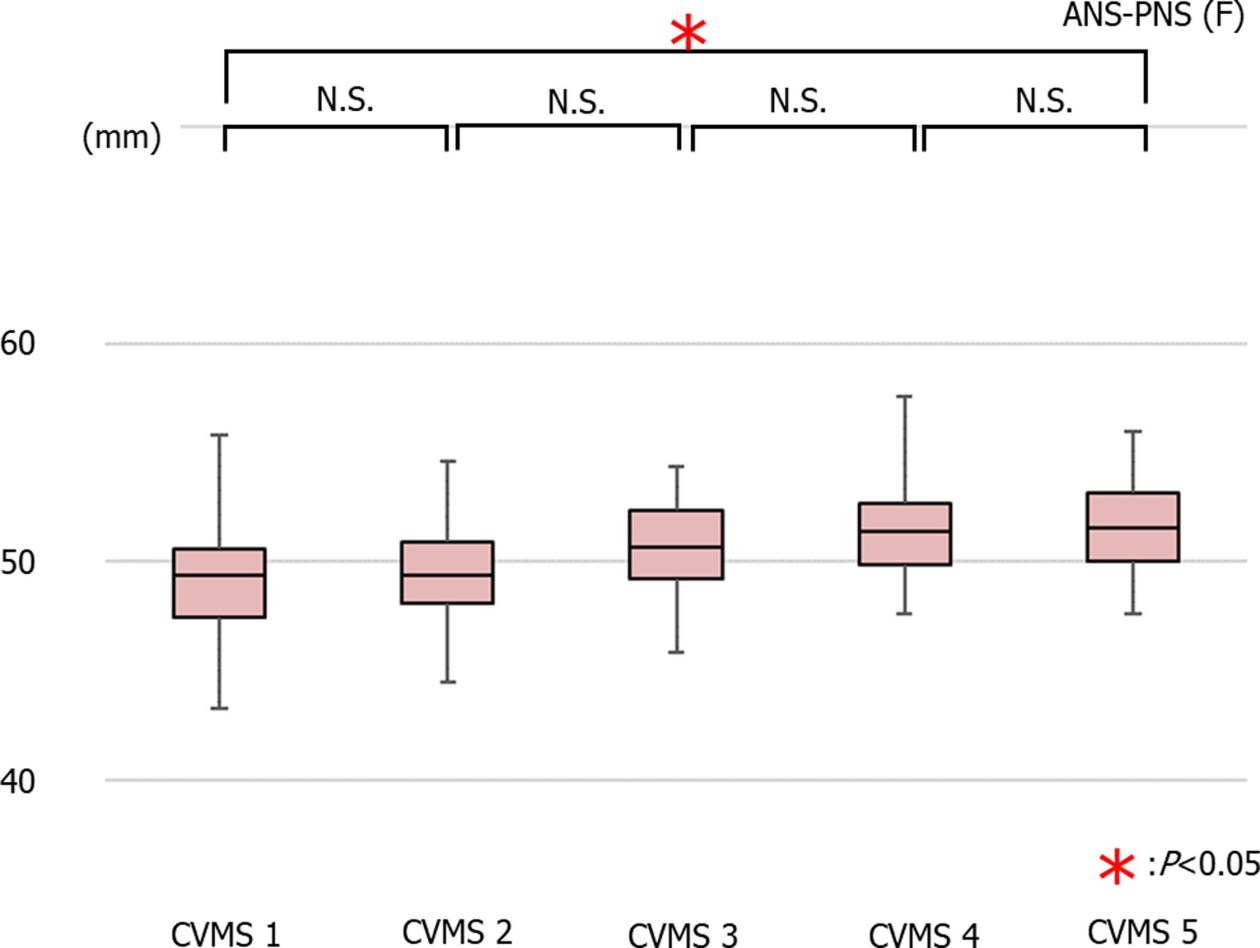
The average values of ANS-PNS in females (*p<0.05).

The average values of ANS-PNS in females were 49.10 ± 2.61 mm in CVMS I, 49.46 ± 2.45 mm in CVMS II, 50.66 ± 2.24 mm in CVMS III, 51.56 ± 2.34 mm in CVMS IV, and 51.53 ± 2.13 mm in CVMS V. There was no significant decrease in the ANS-PNS value across all groups; however, there was a significant decrease in this value between CVMS I and CVMS V (Fig 3).

**Fig 4:**
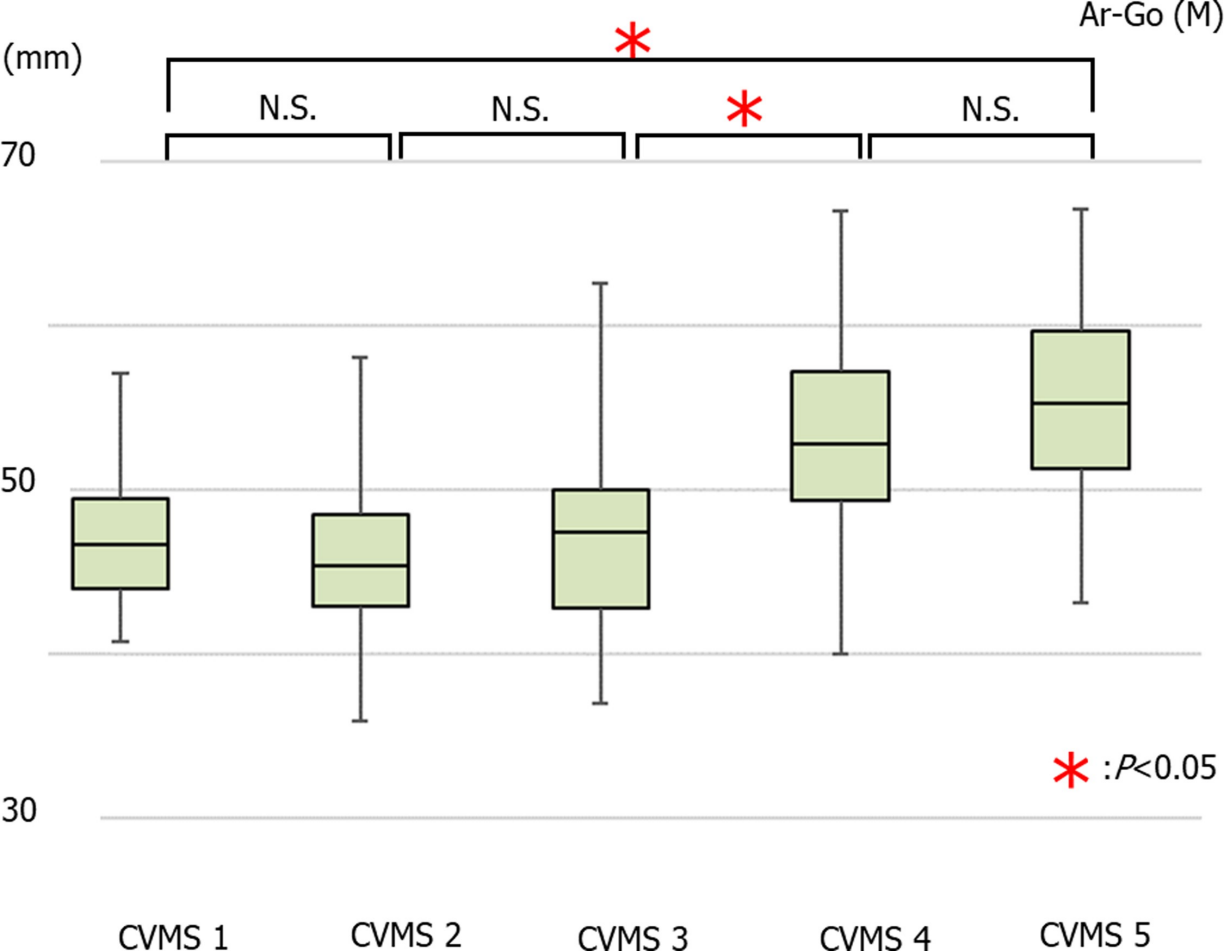
The average values of Ar-Go in males (*p<0.05).

The average values of Ar-Go in males were 46.80 ± 3.76 mm in CVMS I Group, 45.55 ± 4.88 mm in CVMS II, 46.93 ± 5.16 mm in CVMS III, 53.15 ± 6.44 mm in CVMS IV, and 54.94 ± 5.87 mm in CVMS V. There was a significant increase in the Ar-Go value in CVMS I compared to those in CVMS IV and V, in CVMS II compared to those in CVMS IV and V, and in CVMS III compared to those in CVMS IV and V (Fig 4).

**Fig 5:**
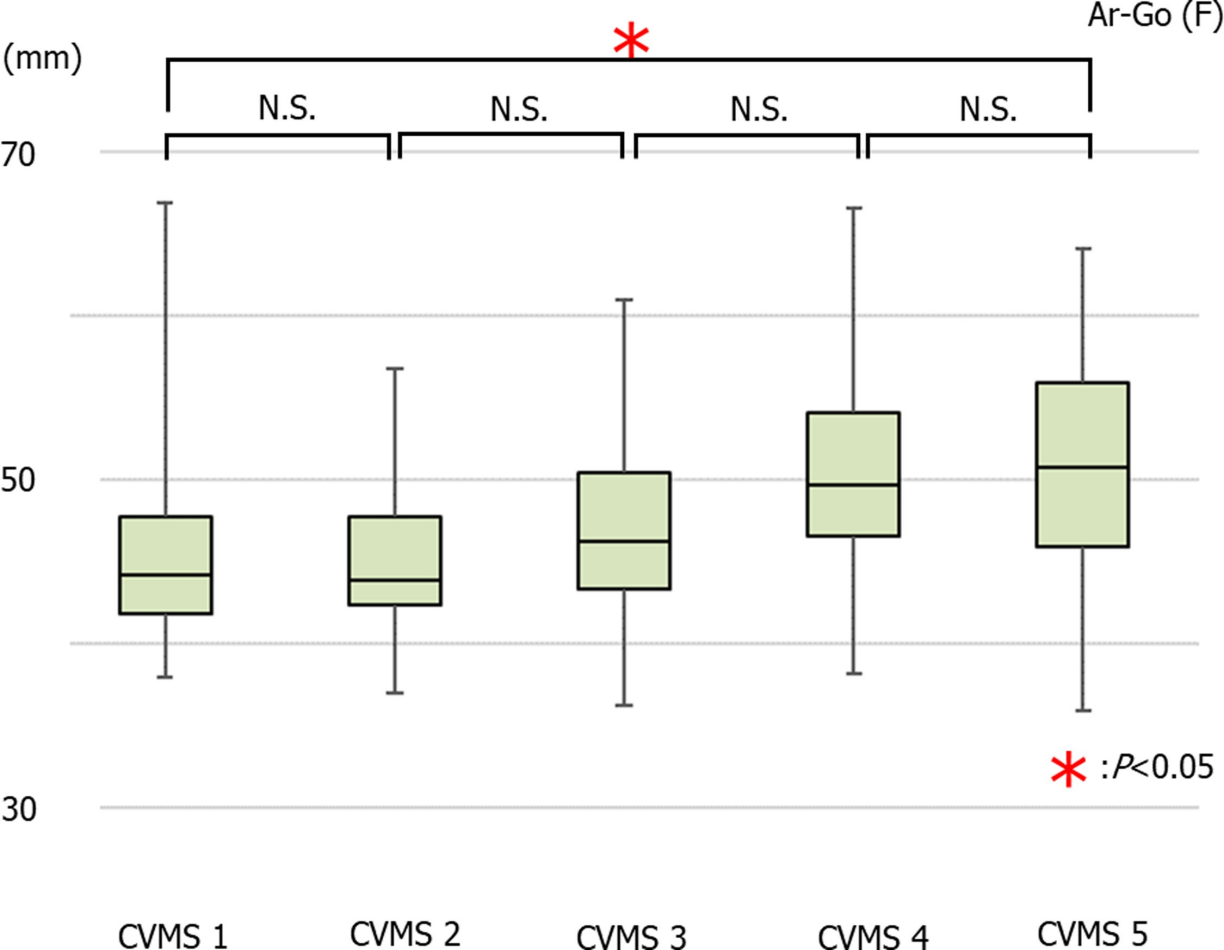
The average values of Ar-Go in females (*p<0.05).

The average values of Ar-Go in females were 45.36 ± 5.24 mm in CVMS I Group, 45.11 ± 4.56 mm in CVMS II, 46.75 ± 5.45 mm in CVMS III, 50.57 ± 6.25 mm in CVMS IV, and 50.76 ± 6.66 mm in CVMS V. There was no significant increase in the Ar-Go value across all groups; however, there was a significant increase in this value between CVMS I and CVMS V (Fig 5).

**Fig 6:**
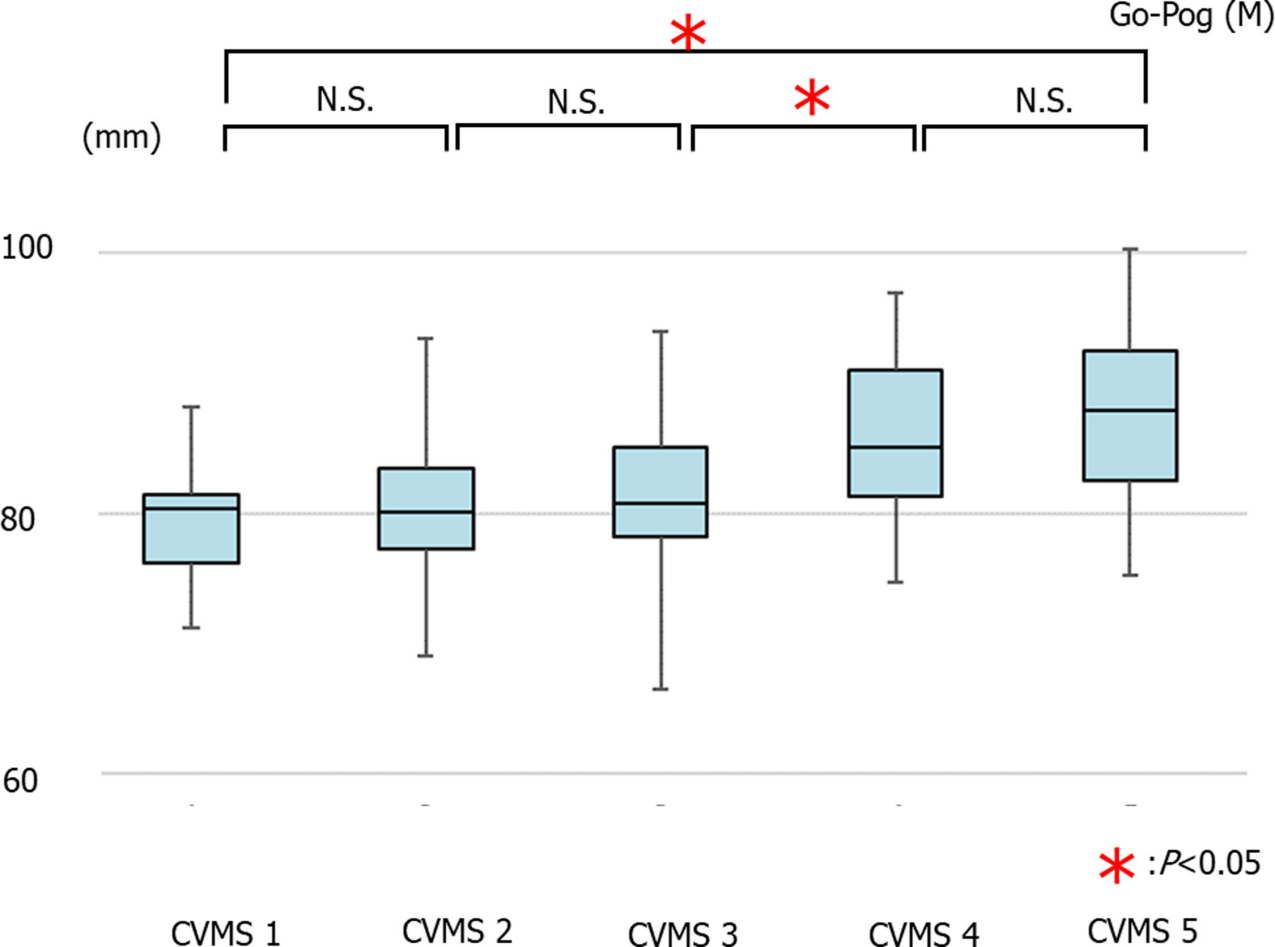
The average values of Go-Pog in males (*p<0.05).

The average values of Go-Pog in males were 79.57 ± 3.97 mm in CVMS I Group, 80.24 ± 5.22 mm in CVMS II, 80.73 ± 5.56 mm in CVMS III, 85.80 ± 6.55 mm in CVMS IV, and 87.97 ± 6.35 mm in CVMS V. There was a significant increase in the Go-Pog value in CVMS I compared to those in CVMS IV and V, in CVMS II compared to those in CVMS IV and V, and in CVMS III compared to those in CVMS IV and V (Fig 6).

**Fig 7:**
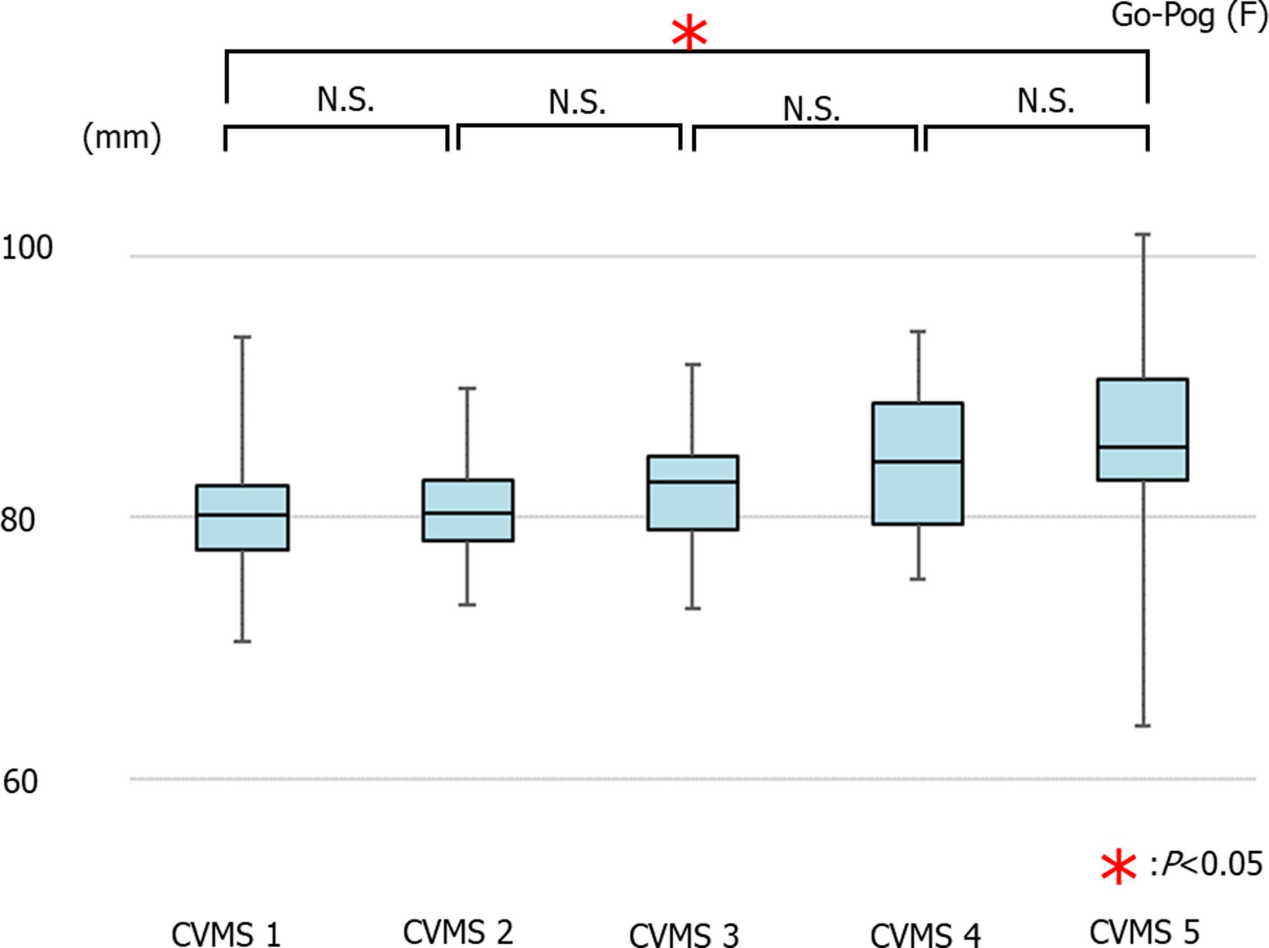
The average values of Go-Pog in females (*p<0.05).

The average values of Go-Pog in females were 80.23 ± 4.96 mm in CVMS I Group, 80.68 ± 4.20 mm in CVMS II, 82.04 ± 4.48 mm in CVMS III, 84.14 ± 5.63 mm in CVMS IV, and 86.66 ± 7.23 mm in CVMS V. There was no significant increase in the Go-Pog value across all groups; however, there was a significant increase between CVMS I and CVMS V (Fig 7).

### Growth pattern comparisons

There is a difference in the timing of large growth between the maxilla and mandible. The timing of the large growth of mandible height and length is the same. There are differences between male and female in the growth patterns of maxillary length and mandibular length and height. (Figs 2–7).

### Reproducibility and reliability

The κ coefficient for the degree of intra-evaluator agreement was 0.89, and the κ coefficient for the degree of inter-evaluator agreement was 0.91, both showing almost perfect agreement.

The measurement error for mandibular height (Ar-Go) was 0.63 mm, as calculated using Dahlberg’s formula, and that for mandibular length (Go-Pog) was 0.67 mm, which was a sufficiently small error.

## Discussion

Identifying the growth spurt of the maxilla and mandible is important for orthodontic treatment during the growth period. However, it is considered difficult to predict this growth because of the differences resulting from the growth spurt of height [10]. At present, carpal roentgens are regarded as the gold standard to assess bone maturation [9]. Indeed, there were several reports on the correlation between the ages determined using carpal bones and the cervical spine [10, 17, 19, 20], while the British Orthodontic Society stated that there was uncertainty using carpal radiography in predicting patient’s adolescent growth spurt [10]. Lateral cephalometric radiographs are indispensable for clinical orthodontic treatment planning and are often taken at the time of the first visit. It is considered clinically valuable to identify growth predictors using these radiographs.

We found that with the growth pattern, the height of the mandible was significantly larger in males than in females. In contrast, there was no significant difference in the mandibular length between males and females. As previously reported in the literature, the height and length of the mandible are significantly larger in males [21–23]. Accordingly, we measured the actual mandibular length to determine the differences between males and females.

A headgear, known as an extra-oral anchorage device, can suppress or redirect the overgrowth of the maxilla by using the head or neck as a fixative anchorage [24]. In the past, it has been reported that using a removable functional appliance in the treatment of maxillary anterior protrusion during the growth period results in slight suppression of maxillary growth [25]. Maxillary length ANS-PNS showed a significant increase from CVMS II to CVMS III in males, revealing the appearance of maxillary growth spurts. Therefore, it is necessary to predict growth tendency via the growth patterns during diagnosis and thereafter.

In this study, the mandibular height (Ar-Go) and length (Go-Pog) were increased in the period between the CVMS III and CVMS IV. In patients with mandibular prognathism, orthodontists try to achieve normal overbite and overjet with a skeletal discrepancy in dental compensation. However, a normal overbite and overjet cannot be constructed by dental compensation alone when a large skeletal discrepancy is present. To correct the anteroposterior jaw position and realign the intermaxillary relation with consideration for the mandible (toward the tendency of elimination of dental compensation) after tooth movement, orthognathic surgery can be used to achieve an adequate maxillo-mandibular relation by splitting the maxillo-mandibular jaw bone in combination with orthodontic treatment [26, 27]. Specifically, the decompensation direction in maxillary and mandibular anterior teeth revealed the opposite from dental compensation in non-surgery mandibular prognathism cases. Orthodontists establish a treatment plan in accordance with the degree of skeletal discrepancy. Nevertheless, if the mandibular spurt is misread, the construction of a normal overbite and overjet cannot be achieved by orthodontic treatment alone. Mistakes in treatment decisions can lead to complications, such as prolonged treatment and root resorption. Moreover, in cases of maxillary prognathism with mandibular retraction, a good intermaxillary relationship can be obtained by inducing the anterior growth of the mandible. Therefore, it was suggested that it is desirable to initiate treatment during CVMS III in males when inducement of mandibular growth is needed. In addition, mandibular prognathism during the growth period may be managed using a combination of orthodontic and surgical treatment when mandibular growth is remarkable [18, 20]. Previous reports have indicated that controlling mandibular growth with functional orthopedic appliance devices is effective in growing patients with skeletal discrepancy [28]. In cases where the observation of mandibular growth is needed, it is considered difficult to determine the appropriate timing for treatment initiation based on the CVMS III findings. Both CVMS and chronological ages should be considered to anticipate future growth potential. It is advisable to initiate treatment during CVMS IV, which corresponds to the plateau of mandibular growth. The confirmation of the specific CVMS on lateral cephalometric radiographs thus facilitates orthodontic treatment planning.

Compared to males, females were not acknowledged as having a relatively obvious growth spurt of maxilla and mandible. A previous study reported that Japanese females revealed a clarified presentation of growth spurt [29]. For orthodontic treatment during the growth period in females, it is considered eligible/advisable to determine a treatment plan considering the difference in growth between females.

Previous research has demonstrated that CVM can be easily evaluated on lateral cephalometric radiographs, and this measure is adequate for predicting the adolescent growth spurt for mandibular growth [28]. Nevertheless, there are reports of this method lacking reproducibility among evaluators [30]. Numerous experienced orthodontists do not measure the shape of the cervical vertebrae before initiating the treatment, which may be explained by a possible lack of skills for tracing and measuring the cervical bone. However, in our research, the weighted kappa coefficient showed an intra-evaluator reproducibility of 0.89 and an inter-evaluator reproducibility of 0.91, both of which were considered almost perfect. Additionally, according to evidence, CVM evaluation can be inaccurate due to a rotation error [31]. It is worth noticing that computed tomography (CT) had been performed without stabilizing the head with the ear rod in the related study. Accordingly, lateral cephalometric radiographs were used to determine CVM in our study. In lateral cephalometric radiographs, rotation of the horizontal and vertical planes is regulated by ear rods, and rotation errors are significantly minimized. Additionally, the x-ray tube is as long as the patient’s midsagittal plane, and the films are always stabilized at a certain distance. Thus, the lateral cephalometric radiographs are standardized, and it is widely known that their reproducibility is relatively high with fewer errors. Numerous clinicians, including orthodontists, are aware of the changes in the shape and size of the cervical spine that occur with growth and that lateral cephalometric radiographs reflect the growth and age of the cervical spine. Furthermore, previous studies using CT in orthopedics have reported a morphological change in the cervical spine with age [32, 33]. Appropriate treatment of patients based on a prediction of the mandibular growth, preferably without additional radiational exposure, is recognized as a priority. We believe that it is particularly important for orthodontists to study the growth of the cervical spine for improved treatment outcomes.

The limitations of this study included its cross-sectional design and inability to follow the continuing growth in individuals. Even though the capability of a cross-sectional study surveying a large subject number of participants and its insensitivity to the differences between individuals, we highlighted the compatibility of this method in our study with the calculation of standard value [34, 35]. Ideally, a longitudinal survey would be preferable. Nonetheless, previous research has exhibited its reproducibility and statistical analysis [17, 34, 35]. It is difficult to observe the original growth of the maxillary and mandibular in patients undergoing orthodontic treatment because their maxilla-mandibular growth could be modified by the treatment. Moreover, it is ethically difficult in modern times to take cephalometric radiographs only for research purposes in patients who do not undergo orthodontic treatment. We reasoned that it was important to grasp the trends from both sides in both the cross-sectional survey and longitudinal survey.

## Conclusion

The standard values of mandibular height and length and maxillary length were calculated, and the mandible and maxilla growth patterns in Japanese were clarified according to sex. The CVMS is a useful aid for growth assessment in patients undergoing orthodontic treatment.

## Supporting information

S1 File (PDF)
