# Supplementary material for: Evaluation of maxillary and mandibular growth patterns with cephalometric analysis based on cervical vertebral maturation: A Japanese cross-sectional study
Source: PLoS One. 2022 Apr 6;17(4):e0265272. doi: 10.1371/journal.pone.0265272 (PMC8985984; doi:10.1371/journal.pone.0265272)
Supplement: S1 File — (PDF) [file pone.0265272.s001.pdf]

	Sex	CVM	暦年齢	Y	SNA	SNB	ANB	MP	Ar-Go(m	Go-Pog[mm	ANS-PNS
1	F	1	117	9.75	78.40	75.00	3.40	30.60	38.90	72.60	51.56
2	F	1	112	9.33	83.10	74.60	8.50	35.40	41.80	77.90	53.87
3	F	1	105	8.75	78.50	68.50	10.00	36.30	40.30	79.30	49.82
4	F	1	119	9.92	83.20	75.30	7.90	38.50	40.20	81.60	51.36
5	F	1	119	9.92	87.70	87.00	0.70	18.50	52.60	88.00	51.69
6	F	1	99	8.25	82.30	77.80	4.50	35.80	46.10	71.60	48.31
7	F	1	96	8.00	80.40	75.30	5.10	29.20	44.40	82.10	49.48
8	F	1	100	8.33	82.40	75.20	7.20	29.40	42.00	80.00	50.34
9	F	1	124	10.33	79.90	76.20	3.70	31.30	47.20	76.90	50.58
10	F	1	113	9.42	79.30	70.40	8.90	35.50	41.10	78.60	50.03
11	F	1	116	9.67	79.80	72.00	7.80	39.00	42.10	73.20	52.40
12	F	1	104	8.67	85.20	77.30	7.90	28.20	47.70	82.00	47.52
13	F	1	75	6.25	84.80	76.00	8.80	30.80	42.20	74.80	45.80
14	F	1	104	8.67	81.60	74.80	6.80	36.80	38.00	87.70	46.97
15	F	1	102	8.50	84.70	82.60	2.10	19.40	53.50	86.10	49.46
16	F	1	113	9.42	83.80	78.00	5.80	26.80	47.50	82.30	50.45
17	F	2	105	8.75	77.20	79.50	-2.30	26.00	42.84	73.50	44.82
18	F	3	106	8.83	80.50	72.00	8.50	29.00	36.21	83.40	46.38
19	F	1	89	7.42	80.00	71.50	8.50	20.70	43.30	75.00	47.88
20	F	3	96	8.00	82.50	78.00	4.50	27.50	42.49	78.80	50.14
21	F	3	136	11.33	79.00	77.00	2.00	26.00	43.96	84.60	49.79
22	F	3	92	7.67	83.00	83.50	-0.50	34.00	44.42	78.50	52.37
23	F	3	106	8.83	84.50	78.00	6.50	26.50	45.00	74.00	54.04
24	F	3	135	11.25	77.00	77.00	0.00	29.50	42.74	79.50	50.90
25	F	3	104	8.67	80.57	78.92	1.65	22.61	47.67	82.40	50.12
26	F	3	116	9.67	83.06	78.23	4.83	24.07	46.23	82.90	47.36
27	F	2	101	8.42	74.23	67.95	6.28	39.72	42.54	75.10	51.85
28	F	5	224	18.67	82.40	86.40	-4.00	25.90	64.10	64.10	47.77
29	F	2	119	9.92	82.90	73.80	9.10	25.20	55.20	82.10	52.99
30	F	5	195	16.25	82.30	79.00	3.30	33.70	59.70	89.40	51.57
31	F	3	129	10.75	79.00	73.50	5.50	33.00	39.50	77.40	51.02
32	F	2	146	12.17	82.50	85.50	-3.00	22.00	41.51	89.40	47.75
33	F	4	216	18.00	85.20	85.70	-0.50	23.60	62.20	91.80	53.48
34	F	3	120	10.00	79.50	76.00	3.50	26.50	41.34	83.50	51.49
35	F	3	128	10.67	90.66	84.93	5.73	27.96	37.98	79.30	53.08
36	F	3	119	9.92	74.12	75.16	-1.00	37.53	39.68	73.00	45.82
37	F	4	195	16.25	82.50	80.00	2.50	26.00	49.60	94.20	49.85
38	F	3	117	9.75	81.00	74.00	7.00	35.00	41.50	76.80	51.84
39	F	3	94	7.83	84.00	76.00	8.00	28.50	44.00	75.50	50.30
40	F	2	113	9.42	81.00	79.00	2.00	30.00	39.00	76.70	49.41
41	F	3	157	13.08	83.90	78.80	5.10	17.20	50.90	85.50	54.05
42	F	2	100	8.33	87.00	85.00	2.00	31.00	37.00	84.50	44.49
43	F	3	132	11.00	82.00	79.00	3.00	28.00	46.17	80.60	49.25
44	F	3	121	10.08	79.50	80.50	-1.00	20.00	42.00	86.50	46.90
45	F	4	187	15.58	80.90	82.00	-1.10	28.10	59.30	93.50	48.55
46	F	4	197	16.42	78.80	72.00	6.80	33.20	38.20	84.10	52.04
47	F	5	223	18.58	76.20	71.00	5.20	35.90	52.80	83.60	47.65
48	F	3	130	10.83	79.90	75.50	4.40	19.10	47.60	87.70	52.48
49	F	4	198	16.50	80.00	79.00	1.00	29.70	54.00	75.30	49.53
50	F	2	117	9.75	80.80	80.90	-0.10	41.20	39.50	79.90	48.12
51	F	2	135	11.25	82.50	77.80	4.70	25.20	43.60	79.00	49.39
52	F	4	158	13.17	82.20	83.40	-1.20	32.40	56.00	82.40	47.85
53	F	3	158	13.17	84.10	80.10	4.00	31.30	39.30	76.60	48.98
54	F	5	229	19.08	81.80	78.70	3.10	34.10	42.20	84.90	53.05
55	F	2	133	11.08	80.00	77.70	2.30	27.90	41.90	78.50	45.71
56	F	5	231	19.25	76.40	74.30	2.10	21.60	56.10	82.10	53.19
57	F	2	139	11.58	82.10	76.00	6.10	21.60	42.80	77.90	51.36
58	F	4	177	14.75	70.30	66.00	4.30	28.40	59.40	84.90	49.89
59	F	5	143	11.92	79.40	76.40	3.00	32.40	48.90	84.90	50.42
60	F	3	129	10.75	78.60	84.00	-5.40	29.20	52.20	91.70	47.89
61	F	5	239	19.92	83.20	78.70	4.50	25.80	53.10	83.40	55.70
62	F	5	223	18.58	81.60	81.30	0.30	15.20	55.20	92.70	50.67
63	F	2	118	9.83	84.40	81.60	2.80	29.90	47.70	80.10	50.72
64	F	2	131	10.92	83.50	77.80	5.70	22.80	45.20	78.30	52.71
65	F	4	190	15.83	78.80	74.80	4.00	25.30	45.70	87.60	53.31
66	F	4	130	10.83	78.80	75.60	3.20	36.20	49.00	90.70	49.88
67	F	5	225	18.75	78.60	72.90	5.70	44.10	35.90	85.80	51.72
68	F	5	186	15.50	84.40	84.50	-0.10	35.10	46.10	97.60	51.62
69	F	3	129	10.75	82.70	79.50	3.20	25.80	47.70	86.30	53.90
70	F	2	120	10.00	77.10	73.30	3.80	31.20	41.80	73.30	45.51
71	F	3	188	15.67	88.40	88.30	0.10	30.70	54.20	79.10	53.67
72	F	5	244	20.33	81.90	73.70	8.20	39.50	42.00	87.40	56.03
73	F	4	118	9.83	78.00	72.60	5.40	36.80	49.70	88.20	50.29
74	F	4	170	14.17	77.20	75.30	1.90	31.40	53.20	87.60	52.06
75	F	2	118	9.83	78.20	72.40	5.80	35.30	42.10	81.50	49.29
76	F	2	109	9.08	78.80	73.00	5.80	32.80	42.40	76.10	47.92
77	F	1	144	12.00	77.70	76.60	1.10	26.90	49.70	81.10	47.33
78	F	2	119	9.92	73.20	72.60	0.60	29.70	43.40	89.90	46.22
79	F	2	132	11.00	84.80	78.30	6.50	30.50	48.10	80.50	49.40
80	F	5	222	18.50	73.00	69.00	4.00	32.90	49.50	82.50	50.07
81	F	4	169	14.08	72.10	69.70	2.40	35.00	41.30	77.10	53.96
82	F	4	175	14.58	81.60	74.60	7.00	30.30	40.80	80.20	52.39
83	F	4	181	15.08	79.20	74.60	4.60	36.20	48.30	76.00	51.33
84	F	5	120	10.00	87.00	74.90	12.10	42.90	42.40	84.80	48.93
85	F	4	233	19.42	81.50	74.00	7.50	31.90	49.60	78.50	57.61
86	F	4	108	9.00	85.20	81.80	3.40	29.00	40.90	91.40	50.62
87	F	4	201	16.75	78.00	76.60	1.40	36.50	52.10	88.80	52.53
88	F	4	201	16.75	82.80	74.30	8.50	33.60	52.50	79.50	56.36
89	F	5	212	17.67	81.00	81.10	-0.10	16.80	56.00	87.60	49.61
90	F	5	235	19.58	88.20	84.30	3.90	33.90	60.70	88.10	50.70
91	F	4	215	17.92	80.70	73.20	7.50	21.90	52.10	76.60	54.86
92	F	2	80	6.67	74.80	73.70	1.10	40.90	50.20	85.90	46.30
93	F	5	238	19.83	80.90	75.20	5.70	39.10	41.90	74.10	53.18
94	F	3	120	10.00	83.10	78.20	4.90	29.10	50.30	83.10	50.01
95	F	4	167	13.92	78.80	73.80	5.00	28.10	54.90	84.90	51.68
96	F	2	102	8.50	86.00	80.20	5.80	18.50	47.40	80.10	54.26
97	F	2	205	17.08	83.90	77.80	6.10	20.50	52.20	75.50	51.85
98	F	2	222	18.50	84.60	76.20	8.40	26.70	48.90	85.60	54.65
99	F	5	144	12.00	80.00	74.50	5.50	45.10	49.80	96.00	49.82
100	F	2	93	7.75	81.50	79.90	1.60	23.90	47.60	87.60	47.70
101	F	5	224	18.67	81.60	79.00	2.60	25.70	45.50	90.30	52.17
102	F	5	180	15.00	81.00	80.60	0.40	22.10	53.20	90.20	53.30
103	F	4	189	15.75	79.40	81.90	-2.50	29.90	59.30	90.80	49.90
104	F	4	76	6.33	78.30	76.80	1.50	20.00	51.40	85.70	50.31
105	F	4	247	20.58	76.50	75.30	1.20	27.00	53.20	90.30	52.66
106	F	2	119	9.92	81.90	78.90	3.00	32.30	46.80	75.30	49.72
107	F	4	190	15.83	80.80	75.30	5.50	23.00	46.50	78.80	51.40
108	F	4	168	14.00	83.30	76.80	6.50	22.00	58.80	86.90	51.14
109	F	5	248	20.67	82.00	77.60	4.40	32.30	52.10	85.00	54.24
110	F	5	105	8.75	79.00	80.50	-1.50	35.60	56.70	97.30	50.13
111	F	5	130	10.83	82.10	78.40	3.70	39.20	44.00	78.70	49.61
112	F	4	130	10.83	73.90	70.40	3.50	31.20	44.80	80.60	51.65
113	F	5	233	19.42	84.00	84.00	0.00	20.50	59.80	91.90	53.20
114	F	2	129	10.75	77.10	71.10	6.00	27.60	43.90	75.00	46.65
115	F	2	241	20.08	83.90	77.90	6.00	27.60	42.40	79.10	49.48
116	F	2	140	11.67	84.20	83.10	1.10	9.10	56.70	82.00	50.17
117	F	2	152	12.67	81.20	76.80	4.40	33.80	47.70	82.60	50.42
118	F	5	105	8.75	80.70	77.00	3.70	26.70	46.00	79.80	53.91
119	F	5	220	18.33	76.80	75.70	1.10	26.90	53.80	85.50	53.55
120	F	1	106	8.83	82.30	78.20	4.10	27.00	46.00	77.60	48.60
121	F	4	238	19.83	81.30	76.80	4.50	27.60	46.10	79.80	51.35
122	F	2	96	8.00	76.00	74.50	1.50	19.00	40.70	82.80	49.10
123	F	5	249	20.75	79.50	79.60	-0.10	36.10	50.70	91.50	51.80
124	F	5	236	19.67	84.30	82.10	2.20	26.70	57.40	85.40	52.00
125	F	5	227	18.92	76.70	73.70	3.00	32.40	46.20	84.80	50.49
126	F	1	134	11.17	81.60	77.80	3.80	27.40	47.80	80.10	55.84
127	F	1	161	13.42	84.40	78.90	5.50	23.50	50.30	79.00	52.36
128	F	1	90	7.50	84.10	78.40	5.70	26.60	51.40	84.00	50.33
129	F	2	128	10.67	76.50	71.60	4.90	38.30	45.90	77.20	52.43
130	F	1	128	10.67	82.20	77.70	4.50	29.40	41.80	76.10	50.84
131	F	1	83	6.92	81.50	78.20	3.30	32.40	43.00	81.30	50.17
132	F	1	131	10.92	84.50	76.90	7.60	35.20	40.20	86.10	48.34
133	F	1	101	8.42	83.40	76.70	6.70	28.30	41.70	70.50	48.20
134	F	5	139	11.58	79.00	73.70	5.30	27.40	50.80	92.60	52.57
135	F	1	179	14.92	84.10	77.50	6.60	24.50	51.40	80.40	49.63
136	F	1	144	12.00	77.10	74.60	2.50	37.20	46.00	79.30	45.44
137	F	5	152	12.67	80.20	75.10	5.10	36.50	47.90	82.50	50.11
138	F	2	103	8.58	77.70	76.60	1.10	27.50	45.80	80.50	48.66
139	F	1	110	9.17	79.00	76.30	2.70	27.10	48.60	88.10	48.83
140	F	2	141	11.75	80.80	81.20	-0.40	24.20	51.10	86.60	52.38
141	F	3	184	15.33	83.40	79.80	3.60	30.50	49.70	85.20	51.30
142	F	2	130	10.83	84.00	81.20	2.80	29.50	43.90	82.90	48.96
143	F	4	211	17.58	82.40	80.10	2.30	23.80	56.60	88.80	53.02
144	F	3	159	13.25	81.80	83.20	-1.40	17.80	60.90	88.80	50.77
145	F	3	122	10.17	82.90	81.20	1.70	30.20	47.30	81.50	50.67
146	F	3	124	10.33	82.10	78.00	4.10	24.80	52.00	83.50	50.39
147	F	4	236	19.67	75.50	71.40	4.10	28.90	49.70	81.50	54.07
148	F	3	123	10.25	82.90	78.70	4.20	27.60	51.00	83.20	54.35
149	F	3	250	20.83	82.20	76.50	5.70	20.90	50.80	83.80	51.49
150	F	4	200	16.67	77.10	71.10	6.00	28.20	53.70	80.90	51.75
151	F	2	137	11.42	79.20	76.40	2.80	35.90	39.80	79.60	49.01
152	F	5	208	17.33	83.20	90.70	-7.50	29.00	55.80	100.80	50.02
153	F	2	151	12.58	91.40	86.60	4.80	30.80	51.00	86.80	48.58
154	F	2	147	12.25	79.10	75.40	3.70	34.60	44.60	82.80	48.51
155	F	5	226	18.83	80.50	87.20	-6.70	31.20	55.40	89.90	47.62
156	F	2	134	11.17	83.20	77.90	5.30	21.90	52.60	78.60	51.76
157	F	2	132	11.00	78.80	76.80	2.00	32.30	43.20	80.80	50.15
158	F	4	155	12.92	79.30	77.80	1.50	35.10	46.60	85.10	48.79
159	F	5	195	16.25	78.30	83.40	-5.10	27.00	50.30	96.10	52.08
160	F	3	132	11.00	80.00	76.60	3.40	24.70	43.50	82.60	47.22
161	F	3	127	10.58	82.30	79.60	2.70	21.40	57.00	91.40	50.31
162	F	4	195	16.25	80.20	78.70	1.50	23.40	46.50	84.30	50.36
163	F	3	233	19.42	83.30	80.10	3.20	25.80	56.70	80.70	49.73
164	F	3	165	13.75	83.70	80.10	3.60	28.80	49.00	85.30	49.07
165	F	2	87	7.25	80.60	74.70	5.90	31.70	42.60	81.70	49.53
166	F	3	164	13.67	73.00	70.50	2.50	33.90	44.80	80.50	51.49
167	F	2	123	10.25	76.90	73.30	3.60	36.00	38.60	78.50	49.70
168	F	4	188	15.67	76.60	74.30	2.30	26.40	48.10	77.40	52.60
169	F	4	178	14.83	76.50	72.60	3.90	28.70	42.30	79.30	50.85
170	F	3	165	13.75	80.50	77.90	2.60	24.30	47.70	75.40	51.77
171	F	4	187	15.58	85.60	79.80	5.80	34.40	46.90	78.30	56.92
172	F	3	140	11.67	78.30	76.50	1.80	24.80	43.80	79.30	48.20
173	F	4	159	13.25	83.80	79.20	4.60	23.60	50.00	75.80	50.42
174	F	5	200	16.67	83.30	84.30	-1.00	38.70	50.70	101.60	53.45
175	F	1	83	6.92	78.20	76.30	1.90	33.90	41.00	80.40	44.00
176	F	4	190	15.83	72.40	74.00	-1.60	24.70	44.80	80.60	52.67
177	F	1	72	6.00	88.30	81.10	7.20	23.40	42.60	85.10	47.90
178	F	1	237	19.75	85.60	84.30	1.30	17.00	66.90	93.80	51.87
179	F	1	161	13.42	78.20	75.30	2.90	29.80	40.90	78.00	49.34
180	F	1	76	6.33	86.20	84.00	2.20	26.30	48.00	81.00	46.28
181	F	1	231	19.25	83.00	84.70	-1.70	27.80	44.00	81.80	47.33
182	F	1	110	9.17	79.60	79.50	0.10	25.40	43.30	79.90	45.66
183	F	2	91	7.58	89.20	80.90	8.30	35.70	44.30	83.30	50.82
184	F	1	78	6.50	75.10	73.90	1.20	33.90	44.90	77.10	43.26

	Sex	CVM	暦年齢	Y	SNA	SNB	ANB	MP	Ar-Go(m	Go-Pog[	ANS-PNS
1	M	1	111	9.25	81.00	76.80	4.20	32.00	42.10	81.60	48.16
2	M	1	91	7.58	81.50	78.60	2.90	26.80	47.90	77.60	50.65
3	M	1	118	9.83	81.90	74.50	7.40	24.10	42.00	78.40	55.44
4	M	1	119	9.92	77.50	76.00	1.50	31.10	41.60	75.20	54.90
5	M	1	129	10.75	79.80	73.50	6.30	35.80	41.40	80.70	50.05
6	M	1	110	9.17	85.20	84.70	0.50	26.20	49.50	78.30	46.88
7	M	1	101	8.42	81.00	72.90	8.10	37.50	44.00	80.70	48.50
8	M	1	105	8.75	77.90	77.10	0.80	37.80	40.70	76.70	52.72
9	M	1	96	8.00	78.30	74.80	3.50	32.70	46.30	88.10	46.01
10	M	1	137	11.42	78.10	74.70	3.40	26.30	49.00	77.90	52.14
11	M	1	125	10.42	83.60	78.70	4.90	30.10	44.70	75.10	52.37
12	M	1	125	10.42	82.50	79.70	2.80	32.70	50.20	88.00	52.70
13	M	1	86	7.17	74.20	76.90	-2.70	27.70	48.20	78.70	46.44
14	M	4	87	7.25	86.00	79.00	7.00	26.00	40.00	87.90	49.39
15	M	3	125	10.42	84.04	79.73	4.31	26.95	42.18	79.70	49.95
16	M	3	119	9.92	80.50	75.00	5.00	23.00	41.96	72.80	52.20
17	M	5	84	7.00	85.30	84.80	0.50	22.80	61.40	94.70	60.77
18	M	3	122	10.17	75.00	70.00	5.00	44.00	39.96	85.40	48.41
19	M	3	136	11.33	81.71	79.10	2.61	24.35	46.73	78.70	55.59
20	M	4	215	17.92	81.90	79.90	2.00	17.20	63.40	96.40	57.49
21	M	4	213	17.75	79.70	75.10	4.60	26.80	53.70	84.40	54.63
22	M	3	128	10.67	87.50	81.50	6.00	31.50	40.00	87.90	50.45
23	M	4	223	18.58	79.40	82.70	-3.30	29.60	60.80	82.10	56.23
24	M	2	128	10.67	80.00	75.00	5.00	30.00	45.37	75.20	49.85
25	M	4	189	15.75	79.20	77.40	1.80	30.20	52.20	88.90	55.23
26	M	3	127	10.58	83.00	79.00	4.00	26.00	43.00	80.80	56.67
27	M	3	130	10.83	76.50	74.50	2.00	28.00	36.97	84.10	52.71
28	M	3	152	12.67	74.95	71.76	3.19	30.29	46.96	73.20	49.36
29	M	4	187	15.58	81.60	82.90	-1.30	32.00	58.30	82.50	57.16
30	M	2	131	10.92	83.50	82.00	1.50	25.00	46.00	81.20	49.89
31	M	2	98	8.17	79.20	74.10	5.10	32.90	36.98	77.60	46.88
32	M	3	134	11.17	77.90	76.00	1.90	25.40	41.65	74.40	50.36
33	M	3	138	11.50	81.80	78.10	3.70	20.60	54.50	87.10	55.38
34	M	3	124	10.33	87.00	79.00	8.00	23.00	47.00	88.80	49.32
35	M	2	112	9.33	79.50	80.00	-0.50	19.50	49.50	79.70	51.41
36	M	3	142	11.83	79.50	74.00	5.50	28.00	40.00	66.50	53.12
37	M	2	91	7.58	79.00	74.20	4.80	16.20	37.85	72.80	44.19
38	M	3	113	9.42	79.50	76.00	3.50	23.00	45.65	80.40	50.12
39	M	2	95	7.92	83.00	75.50	7.50	32.00	39.45	78.70	48.03
40	M	3	145	12.08	84.00	76.00	8.00	27.50	43.44	81.30	56.63
41	M	2	128	10.67	82.10	77.80	4.30	28.90	45.00	77.50	50.93
42	M	3	118	9.83	84.00	75.00	9.00	30.00	40.70	81.60	52.12
43	M	5	250	20.83	82.30	85.80	-3.50	23.10	57.20	96.20	58.77
44	M	2	98	8.17	80.00	76.50	3.50	37.50	42.60	80.20	50.48
45	M	2	105	8.75	79.00	78.00	1.00	28.50	36.99	78.60	48.10
46	M	3	176	14.67	81.50	74.90	6.60	31.60	51.40	85.50	55.58
47	M	2	85	7.08	81.07	72.78	8.30	28.06	40.57	79.80	50.86
48	M	4	232	19.33	79.10	78.30	0.80	28.20	50.80	76.90	60.34
49	M	1	112	9.33	83.60	79.20	4.40	21.90	51.70	83.60	50.58
50	M	4	151	12.58	78.20	76.10	2.10	25.90	52.60	81.70	53.66
51	M	1	92	7.67	76.80	75.40	1.40	36.50	42.40	80.60	46.93
52	M	3	137	11.42	83.40	79.40	4.00	27.20	46.40	84.80	57.79
53	M	4	203	16.92	80.20	82.40	-2.20	32.50	53.20	81.10	51.23
54	M	1	84	7.00	84.30	87.70	-3.40	24.90	50.70	80.90	41.74
55	M	1	94	7.83	80.20	75.90	4.30	30.70	46.60	86.60	51.11
56	M	1	93	7.75	83.70	81.30	2.40	28.10	49.40	81.10	48.54
57	M	4	181	15.08	77.10	76.80	0.30	36.20	53.30	83.30	49.60
58	M	4	174	14.50	80.70	75.60	5.10	29.60	53.30	81.40	54.68
59	M	3	127	10.58	81.60	76.10	5.50	25.30	48.00	81.30	50.33
60	M	5	173	14.42	84.10	82.00	2.10	30.30	47.20	91.10	62.87
61	M	5	239	19.92	85.20	80.80	4.40	28.50	50.60	91.30	54.85
62	M	3	171	14.25	77.80	73.10	4.70	24.50	48.20	76.50	54.42
63	M	1	114	9.50	76.30	69.60	6.70	28.80	41.30	76.30	52.75
64	M	5	227	18.92	78.40	78.00	0.40	43.80	50.80	75.20	58.51
65	M	1	97	8.08	80.00	77.00	3.00	35.90	43.70	75.80	46.59
66	M	2	121	10.08	80.70	78.40	2.30	25.30	50.30	87.80	55.22
67	M	5	227	18.92	83.30	77.30	6.00	22.20	51.40	86.00	60.00
68	M	4	216	18.00	88.20	89.60	-1.40	19.90	52.50	85.80	55.44
69	M	4	157	13.08	80.30	76.20	4.10	27.40	45.00	87.60	55.61
70	M	5	105	8.75	83.30	78.00	5.30	27.40	50.00	79.30	62.79
71	M	5	194	16.17	77.80	74.00	3.80	33.40	44.40	81.80	56.60
72	M	5	230	19.17	82.30	77.90	4.40	23.10	64.00	89.80	58.00
73	M	4	128	10.67	83.10	78.90	4.20	27.80	51.00	81.80	58.06
74	M	4	189	15.75	74.50	80.80	-6.30	18.80	52.95	86.60	53.17
75	M	5	128	10.67	77.70	70.90	6.80	39.10	45.70	83.00	56.71
76	M	5	211	17.58	83.10	76.60	6.50	37.60	52.20	87.40	58.09
77	M	5	119	9.92	79.00	78.90	0.10	31.50	52.10	81.90	55.37
78	M	5	218	18.17	78.70	73.90	4.80	40.50	51.00	87.30	58.49
79	M	5	220	18.33	84.70	80.10	4.60	24.90	51.60	87.60	54.45
80	M	2	140	11.67	89.50	84.00	5.50	17.80	58.00	91.60	51.75
81	M	2	142	11.83	80.00	74.50	5.50	28.00	43.90	80.50	54.19
82	M	4	223	18.58	82.60	85.10	-2.50	31.90	53.80	96.90	54.78
83	M	4	198	16.50	73.30	75.70	-2.40	30.00	56.80	81.70	53.96
84	M	3	122	10.17	84.60	78.90	5.70	27.30	46.00	82.30	55.02
85	M	1	123	10.25	78.60	73.70	4.90	37.90	44.60	82.70	48.24
86	M	2	115	9.58	74.40	66.30	8.10	38.20	35.90	75.90	49.29
87	M	5	207	17.25	78.10	80.40	-2.30	29.80	59.30	92.90	55.50
88	M	3	157	13.08	80.40	75.40	5.00	27.90	49.90	85.00	56.51
89	M	2	234	19.50	85.90	80.30	5.60	23.00	45.70	81.50	51.79
90	M	2	115	9.58	79.00	74.90	4.10	26.10	40.30	74.20	49.85
91	M	3	150	12.50	83.70	80.00	3.70	25.60	62.60	87.60	57.79
92	M	4	221	18.42	82.40	79.10	3.30	20.10	53.00	87.90	56.10
93	M	3	117	9.75	82.70	79.60	3.10	29.40	48.50	79.90	51.89
94	M	5	234	19.50	78.80	82.40	-3.60	22.60	56.40	100.20	52.60
95	M	4	174	14.50	83.90	79.20	4.70	24.30	48.70	88.20	60.42
96	M	5	243	20.25	78.00	80.60	-2.60	29.90	55.90	94.30	52.19
97	M	4	199	16.58	77.40	72.00	5.40	32.70	43.20	74.70	52.85
98	M	2	139	11.58	82.00	76.60	5.40	22.70	43.70	85.80	53.87
99	M	5	190	15.83	87.50	84.10	3.40	24.70	57.30	90.40	56.78
100	M	2	130	10.83	76.30	70.40	5.90	17.80	46.50	69.10	47.92
101	M	2	131	10.92	82.20	78.10	4.10	25.60	44.00	83.50	52.57
102	M	4	212	17.67	78.40	71.00	7.40	21.80	46.20	77.40	57.62
103	M	2	136	11.33	81.10	78.10	3.00	24.80	49.50	76.70	57.70
104	M	3	146	12.17	83.30	83.50	-0.20	25.20	48.30	85.20	53.58
105	M	4	219	18.25	78.50	81.50	-3.00	25.40	56.80	95.70	56.30
106	M	4	129	10.75	84.40	78.40	6.00	22.10	52.10	78.30	60.16
107	M	2	76	6.33	77.80	77.40	0.40	22.50	44.60	79.90	49.35
108	M	5	217	18.08	79.90	82.90	-3.00	21.80	60.70	88.10	58.27
109	M	5	234	19.50	85.40	86.80	-1.40	33.40	51.90	96.50	58.99
110	M	5	226	18.83	83.70	86.20	-2.50	16.10	67.10	98.60	52.49
111	M	2	151	12.58	83.40	77.30	6.10	23.60	47.80	80.70	53.54
112	M	5	194	16.17	73.50	71.40	2.10	38.20	43.10	80.10	54.46
113	M	4	226	18.83	81.40	82.30	-0.90	24.10	51.80	93.40	55.17
114	M	5	219	18.25	87.20	80.20	7.00	27.80	54.50	82.50	59.84
115	M	5	102	8.50	80.90	75.30	5.60	42.90	44.50	77.90	57.20
116	M	4	236	19.67	82.60	81.00	1.60	34.20	52.20	80.10	53.15
117	M	2	120	10.00	82.10	76.80	5.30	22.90	50.90	73.30	50.44
118	M	5	229	19.08	84.40	93.50	-9.10	25.60	63.40	98.90	54.52
119	M	2	120	10.00	77.20	78.70	-1.50	26.90	47.60	86.00	50.86
120	M	4	197	16.42	79.70	74.70	5.00	38.60	40.50	84.40	48.78
121	M	1	116	9.67	74.50	74.10	0.40	32.50	45.90	75.10	46.80
122	M	2	105	8.75	81.30	77.60	3.70	20.80	45.30	82.70	54.94
123	M	2	145	12.08	80.30	77.70	2.60	27.30	48.50	85.40	50.48
124	M	1	192	16.00	77.40	76.90	0.50	27.00	47.90	78.40	46.48
125	M	3	151	12.58	76.70	70.80	5.90	34.50	50.90	74.10	55.56
126	M	5	225	18.75	83.10	90.00	-6.90	29.60	59.10	89.60	51.99
127	M	1	119	9.92	82.30	82.20	0.10	23.60	50.00	80.70	48.45
128	M	2	202	16.83	80.10	77.50	2.60	19.50	52.60	93.40	50.83
129	M	2	98	8.17	76.70	72.10	4.60	36.40	44.90	80.20	47.80
130	M	2	112	9.33	73.70	71.90	1.80	31.50	43.00	80.70	48.69
131	M	5	230	19.17	80.70	73.90	6.80	30.80	50.30	86.80	56.98
132	M	5	218	18.17	88.20	79.90	8.30	21.40	60.20	88.10	60.59
133	M	5	248	20.67	82.60	76.40	6.20	23.60	52.00	82.40	52.60
134	M	1	247	20.58	74.30	75.50	-1.20	23.90	51.00	80.70	50.33
135	M	2	131	10.92	87.40	85.60	1.80	21.50	52.70	79.40	57.10
136	M	1	105	8.75	82.40	76.30	6.10	30.00	49.20	77.80	49.41
137	M	1	95	7.92	80.10	78.60	1.50	21.20	49.10	75.70	46.98
138	M	3	198	16.50	83.60	79.50	4.10	30.00	45.00	80.30	52.90
139	M	2	124	10.33	81.90	76.00	5.90	26.50	44.30	78.00	51.21
140	M	3	142	11.83	85.20	79.80	5.40	28.60	49.10	81.50	54.00
141	M	1	134	11.17	81.10	76.00	5.10	30.70	42.50	80.10	48.32
142	M	1	162	13.50	78.60	71.90	6.70	34.50	46.00	71.20	50.22
143	M	1	104	8.67	79.20	78.00	1.20	30.60	46.60	81.40	45.82
144	M	4	112	9.33	82.50	76.70	5.80	29.90	44.60	81.60	54.81
145	M	1	112	9.33	80.30	73.50	6.80	33.60	44.00	75.60	49.37
146	M	3	155	12.92	80.50	75.90	4.60	20.20	50.50	80.20	53.14
147	M	2	126	10.50	79.00	72.70	6.30	35.20	39.90	69.70	51.26
148	M	1	248	20.67	80.60	77.20	3.40	19.70	50.00	75.70	48.75
149	M	4	178	14.83	85.50	81.60	3.90	21.60	49.60	88.90	55.79
150	M	4	251	20.92	77.80	77.60	0.20	27.30	63.80	93.20	53.28
151	M	1	179	14.92	81.90	79.50	2.40	32.50	52.10	84.30	51.12
152	M	4	239	19.92	88.40	82.00	6.40	21.10	59.30	89.40	59.97
153	M	4	225	18.75	84.70	88.00	-3.30	20.90	63.40	96.60	57.02
154	M	4	229	19.08	79.20	83.30	-4.10	27.90	67.00	93.00	54.86
155	M	2	107	8.92	82.10	79.80	2.30	21.70	47.70	83.40	53.67
156	M	1	85	7.08	71.30	71.40	-0.10	27.80	43.80	72.80	47.04
157	M	2	244	20.33	75.80	73.80	2.00	32.00	44.50	74.90	46.33
158	M	1	127	10.58	81.40	76.00	5.40	37.20	44.90	80.50	49.98
159	M	4	134	11.17	81.20	77.20	4.00	32.60	48.40	74.80	54.12
160	M	1	90	7.50	80.20	79.50	0.70	26.40	52.90	81.20	47.04
161	M	2	99	8.25	81.40	78.00	3.40	27.70	42.40	78.80	46.72
162	M	1	154	12.83	80.90	76.90	4.00	28.90	48.00	83.30	49.27
163	M	1	116	9.67	85.20	77.70	7.50	26.80	45.90	81.80	50.84
164	M	4	222	18.50	82.00	80.00	2.00	23.50	53.90	93.50	60.79
165	M	2	149	12.42	79.60	78.70	0.90	26.70	47.70	84.70	45.11
166	M	3	147	12.25	77.20	75.90	1.30	27.40	49.80	80.70	53.44
167	M	5	215	17.92	79.40	75.60	3.80	34.70	51.80	78.60	53.69
168	M	5	239	19.92	81.70	78.70	3.00	31.30	62.20	82.20	55.40
169	M	3	154	12.83	80.40	74.80	5.60	39.20	43.00	75.00	53.55
170	M	2	153	12.75	80.10	76.40	3.70	28.90	52.70	86.10	49.83
171	M	3	148	12.33	84.80	81.00	3.80	29.10	48.50	79.30	55.67
172	M	4	235	19.58	75.40	82.30	-6.90	20.80	60.40	79.00	56.40
173	M	5	225	18.75	80.80	86.70	-5.90	37.40	60.70	94.50	52.03
174	M	4	159	13.25	80.90	74.90	6.00	29.90	49.80	77.60	57.46
175	M	1	93	7.75	76.20	72.10	4.10	34.80	57.10	86.30	49.90
176	M	2	158	13.17	83.40	82.40	1.00	30.50	48.40	83.50	52.96
177	M	3	148	12.33	81.90	76.10	5.80	17.20	51.50	75.60	54.75
178	M	5	219	18.25	84.50	82.40	2.10	23.90	59.50	84.30	56.22
179	M	3	176	14.67	87.10	82.40	4.70	31.10	47.80	81.90	51.97
180	M	1	98	8.17	85.60	77.00	8.60	24.40	46.90	75.40	52.77
181	M	5	192	16.00	77.10	78.10	-1.00	44.40	56.20	90.60	52.71
182	M	2	168	14.00	82.00	76.50	5.50	24.80	46.40	76.60	51.88
183	M	4	178	14.83	79.80	72.80	7.00	22.60	48.70	78.80	50.70
184	M	5	229	19.08	84.30	84.20	0.10	36.50	51.80	92.90	52.42

185 F	1	133	11.08	81.20	75.10	6.10	26.30	47.40	82.60	48.29
186 F	5	227	18.92	77.40	70.30	7.10	32.10	40.40	82.80	53.43
187 F	5	234	19.50	78.30	74.20	4.10	18.70	45.50	81.60	49.77
188 F	1	143	11.92	84.40	78.90	5.50	30.20	45.00	83.60	50.75
189 F	1	87	7.25	74.10	69.40	4.70	36.90	43.70	72.40	45.69
190 F	5	219	18.25	84.50	79.90	4.60	28.90	59.40	83.80	51.15
191 F	5	216	18.00	82.80	77.20	5.60	33.00	47.30	76.80	55.28
192 F	5	223	18.58	82.50	84.30	-1.80	26.80	61.50	85.60	48.69
193 F	3	154	12.83	79.30	77.10	2.20	25.30	44.10	83.10	53.03
194 F	3	168	14.00	82.30	79.80	2.50	23.90	52.70	84.10	53.37
195 F	4	211	17.58	83.20	82.90	0.30	29.00	66.50	91.30	48.95
196 F	4	175	14.58	74.00	69.90	4.10	35.60	47.80	83.10	47.60
197 F	4	196	16.33	83.60	88.20	-4.60	28.30	54.30	92.90	48.04
198 F	3	239	19.92	86.10	87.20	-1.10	30.80	49.80	88.40	52.53
199 F	5	214	17.83	81.10	75.00	6.10	33.00	43.50	82.80	51.08
200 F	3	113	9.42	81.00	74.70	6.30	26.60	46.00	77.90	48.98

185 M	3	119	9.92	74.50	71.20	3.30	38.70	41.30	70.60	47.71
186 M	5	242	20.17	78.90	80.70	-1.80	35.90	61.60	85.50	49.11
187 M	5	192	16.00	80.70	81.70	-1.00	37.90	60.10	92.30	48.47
188 M	5	249	20.75	80.90	79.00	1.90	26.90	58.31	78.50	57.31
189 M	5	231	19.25	75.90	80.00	-4.10	26.90	51.80	92.00	54.44
190 M	2	246	20.50	81.80	76.90	4.90	23.10	52.10	84.10	52.63
191 M	3	175	14.58	80.00	78.90	1.10	31.10	52.60	85.90	52.62
192 M	4	195	16.25	84.60	90.30	-5.70	28.10	62.10	90.80	50.54
193 M	4	181	15.08	81.60	77.90	3.70	28.80	46.50	91.40	50.51
194 M	3	189	15.75	75.00	66.20	8.80	26.20	47.90	72.90	52.45
195 M	3	164	13.67	83.30	78.70	4.60	19.30	55.40	93.90	53.37
196 M	3	158	13.17	69.60	64.30	5.30	33.30	40.60	79.80	53.65
197 M	3	162	13.50	79.50	76.10	3.40	31.10	50.10	85.90	54.00
198 M	3	194	16.17	83.60	86.50	-2.90	28.50	53.30	80.70	50.35
199 M	5	224	18.67	77.80	77.30	0.50	26.70	58.20	87.60	52.31
200 M	4	192	16.00	86.90	90.50	-3.60	25.50	60.40	96.10	50.69
